# Barriers to cervical screening and interest in self-sampling among women who actively decline screening

**DOI:** 10.1177/0969141318767471

**Published:** 2018-04-13

**Authors:** Kirsty F Bennett, Jo Waller, Amanda J Chorley, Rebecca A Ferrer, Jessica B Haddrell, Laura AV Marlow

**Affiliations:** 1 Cancer Communication and Screening Group, Department of Behavioural Science and Health, UCL, London, UK; 2 Basic Biobehavioral and Psychological Sciences Branch, Behavioral Research Program, Division of Cancer Control and Population Sciences, National Cancer Institute, Rockville, USA

**Keywords:** Cervical screening, non-attender, decline, informed, intention, barriers, human papillomavirus sampling

## Abstract

**Objectives:**

Understanding why some women actively decline cervical screening could contribute to tailored intervention development. We explored reasons for non-participation in cervical screening among women who had made an active decision not to attend in the future. We also explored interest in human papillomavirus self-sampling.

**Methods:**

In a population-based survey of women in Great Britain, home-based computer-assisted interviews were carried out with screening eligible women. Women reported their intention to attend for screening when next invited. They endorsed predefined barriers to screening and indicated their interest in human papillomavirus self-sampling.

**Results:**

Women who had actively declined screening and those who intended to go but were currently overdue (n=543) were included in this analysis. Women who had made an active decision not to be screened in the future were more likely to endorse the barriers ‘I have other more important things to worry about’ and to perceive screening to be of low relevance based on their sexual behaviour. Most participants (70%) indicated that they would be interested in human papillomavirus self-sampling. Interest in self-sampling was greater among those who reported having had a bad experience of screening in the past, were too busy or embarrassed to attend, or would not want a man to carry out the test.

**Conclusions:**

Women who had made an active decision not to attend screening felt it was of low relevance to them and that they had more important things to worry about. Shifting the perceived cost–benefit ratio for these women by offering human papillomavirus self-sampling might increase screening participation in this group.

## Introduction

In the United Kingdom, women aged 25–64 are regularly invited for cervical screening as part of the NHS Cervical Screening Programme.^1^ The programme is a cost-free, call–recall service, and all women registered with a General Practitioner (GP), regardless of whether they attended screening when previously invited, are invited for screening every three (25- to 49-year-olds) or five years (50- to 64-year-olds). Since the cervical screening programme began in 1988, it has been estimated that up to 5000 cervical cancer deaths a year have been prevented due to screening in England and Wales.^2^ However, the proportion of eligible women being screened has declined from 76% in 2011 to 72% in 2017.^3^ Identifying the reasons why women do not attend for screening is important, to develop effective interventions to improve informed uptake. Studies exploring screening non-attendance using qualitative and quantitative methods suggest a wide range of barriers, including practical barriers such as difficulties arranging appointments,^4^ emotional barriers including embarrassment and fear of what the test might find,^5^ and low perceived risk of cervical cancer.^5–7^

The Precaution Adoption Process Model (PAPM) suggests that individuals move through a series of stages towards participating in a given health behaviour, while acknowledging that some non-participants will actively decide not to participate.^8^ We recently used the PAPM to determine the prevalence of different cervical screening non-participant types.^9^ This research found that most non-participants were aware of screening and had made a decision about future attendance. The majority of these intended to go for screening despite currently being overdue or unscreened, but some had made an active decision not to attend for screening in future (15% of all non-participants). Exploring the reasons why some women have chosen not to attend screening would help to inform the development of tailored interventions for this group. If a woman has made an informed choice not to attend, this is to be respected, but if the decision is based on misconceptions (e.g. about risk), or relates to aspects of the procedure that are perceived as aversive (e.g. the speculum examination) or inconvenient, appropriate interventions could potentially overcome these barriers.

Numerous studies have explored barriers to cervical screening in the United Kingdom^4,5,10^ and beyond,^7,11,12^ but previous research focusing on the reasons why individuals *actively decide* not to attend cervical screening is limited. Blomberg et al.^13^ explored why women did not wish to participate in a population-based cervical screening programme at present, or in the future, in Stockholm, Sweden. Fax messages detailing the reasons for declining screening, and a small number of telephone interviews were analysed qualitatively. A lack of confidence in the benefits of screening, low perceived risk of cervical cancer, and a belief in one’s ability to detect health changes via symptoms were the most commonly described reasons for choosing not to be screened.^13^ The study also found that previous negative experiences of screening, or health care in general, contributed to the decision.^13^ Another Swedish qualitative study found that women who actively chose not to be screened were aware of the benefits of screening, but did not feel that they needed screening because they felt healthy and did not think their personal risk of getting cervical cancer was high.^14^ Low self-esteem, a negative body image, and anticipated discomfort were also discussed, and by choosing not to attend screening, women felt that they could avoid a situation in which they felt vulnerable.^14^ Finally, women commented that they had a number of demands on their time, such as work or childcare, so as long as they felt healthy, screening was not considered a priority.^14^

One way of overcoming some of the practical and emotional barriers to cervical screening may be to offer human papillomavirus (HPV) self-sampling. HPV is a common sexually transmitted infection,^15^ and nearly all cases of cervical cancer can be attributed to an HPV infection.^16^ HPV self-sampling allows women to collect a sample of cells, which can then be sent to a laboratory and tested for HPV. While it is not currently offered by the NHS Cervical Screening Programme, a number of studies have suggested that self-sampling for HPV is acceptable to women,^17–20^ and a recent review found that offering HPV self-sampling increased participation by around 10% in screening non-attenders.^21^ Another study exploring previous barriers to screening, among women who had been overdue for screening but carried out an HPV self-test, found that both practical and emotional barriers had played a role in why they had not attended GP-based screening.^22^

The main aims of the study were to (1) explore self-reported barriers to cervical screening among women who have made an active decision not to attend in the future, compared with those who intended to be screened; and (2) assess whether HPV self-sampling would be acceptable to these women, and whether this alternative method addressed specific barriers to screening.

## Methods

Data were collected as part of a larger survey of non-attendance at cervical screening.^9^ Fieldwork was carried out by a market research agency (TNS) as part of their regular Omnibus survey. Data were collected using face-to-face computer-assisted personal interviews with screening eligible women (i.e. aged 25–64) in Great Britain. Overall, 3112 eligible women took part and provided sufficient data for their future screening intention to be determined. Only women who were classified as non-participants were asked additional questions about their attitudes to screening.

### Measures

*Past screening behaviour*: Past screening behaviour was assessed using three questions. Women who had not either had a hysterectomy or been diagnosed with cervical cancer were asked ‘Have you ever heard of cervical screening, also known as the smear test or Pap test?’ (yes, no, don’t know). Those who responded yes to this question were then asked ‘Have you ever had a cervical screening test?’ (yes, no, don’t know) followed, if applicable, by ‘When was the last time you had a cervical screening test?’ (within the last three years, 3–5 years ago, longer than five years, don’t know). Using these three items and accounting for the participant’s age (which determines their recommended screening interval), women were coded as ‘never screened’, ‘up-to-date’, or ‘overdue’.

*Intention to attend cervical screening in future*: Future intention to be screened was assessed using the question ‘Do you intend to go when next invited?’ (Yes, no, don’t know). Those who responded ‘Yes’ were coded as ‘intenders’ and those who responded ‘no’ were coded as ‘active decliners’.

*Barriers to screening*: Women who were classified as overdue or who did not plan to attend screening in the future were presented with a predefined list of barriers to screening, and the instruction ‘There are many reasons why women don’t go for screening. Do any of these apply to you?’. Sixteen barriers were presented in randomized order (to reduce response bias). The barriers were adapted from previous studies of cervical screening^4,5,23,24^ and were designed to assess a range of reasons why women may not attend screening, including perceived relevance and value, previous experiences, and practical barriers. Women were asked to select as many reasons as applicable. If none of the options were applicable, women could select ‘other’ and type in their reason. Where a woman had selected ‘other’ and written a reason that related to one of the 16 predefined barriers it was recoded as that barrier following discussion between two authors (n = 9).

Six of the 16 barriers (‘I have never been sexually active so I don’t need screening’, ‘I am no longer sexually active so I don’t need screening’, ‘I’ve been with the same partner for a long time so I don’t need screening’, ‘I have only ever been with one partner so I don’t need screening’, ‘I’ve only ever had sex with women so I don’t need screening’, and ‘I currently only have sex with women so I don’t need screening’) related to the perception that cervical screening was of low relevance because of their sexual behaviour. A new variable was derived whereby women endorsing at least one of these six barriers were coded as endorsing ‘Low perceived relevance based on sexual behaviour’.

*Self-sampling:* Interest in self-sampling was assessed using the question ‘In the future, it may be possible for women to do the test themselves at home, using a vaginal swab (similar to a cotton bud). Would you prefer this option?’ (yes definitely, yes probably, probably not, definitely not, don’t know). This was recoded into a binary variable with those responding ‘yes’ (probably or definitely) coded as being interested in self-sampling, and those responding ‘probably not’, ‘definitely not’, or ‘don’t know’ coded as not being interested in self-sampling.

*Sociodemographic variables*: Sociodemographic variables were collected using items designed by TNS or based on the 2011 census. These included age, marital status, working status, and social grade. Social grade represented the occupation of the Chief Income Earner in the household: AB managerial/professional, C1 supervisory, C2 skilled manual, D semi-skilled/unskilled manual, E casual/lowest grade workers.^25^ Ethnicity was assessed using the 2011 census question.^26^ Responses were collapsed into the following: White British or Irish, White Other, South Asian, Black, Mixed, or other ethnic background.

### Analysis

Analyses were carried out using SPSS v23. The PAPM model describes how people first become aware of and engaged with the idea of screening before making a decision about attending and then translating this into action. We were interested in reasons for non-participation in cervical screening among women who had made an active decision about whether to attend in the future (i.e. exploring the post-decision-making part of the PAPM, before intention is translated into action) and have therefore included in the current analyses women who were considered to be ‘intenders’ (were intending to be screened, but currently overdue for screening) or ‘active decliners’ (had decided not to be screened in the future).

Univariate logistic regression models were used to explore whether being an active decliner (versus an intender) was associated with: (i) sociodemographic factors or (ii) specific barriers to screening. Variables that were significant at the p < 0.05 level were entered into a multivariate logistic regression model to explore the adjusted effects.

We used chi-square tests to explore differences in interest in HPV self-sampling between women who were intenders and active decliners, and logistic regression to see if endorsing any particular barriers was associated with interest in HPV self-sampling. As the focus of this paper was the comparison of two subgroups, rather than reporting the group distributions, we used unweighted data.

## Results

Of the 3112 eligible women who completed the survey, 14% (n = 426) were classified as intenders and 4% (n = 117) were classified as active decliners (The remaining women were maintainers (i.e. up to date and intending to be screened in the future), unaware of, unengaged with, or undecided about screening. More detail of the overall distribution of non-participants is reported elsewhere.^7^). Characteristics of the sample in the current analyses and sociodemographic correlates of being an active decliner are shown in Table 1.

**Table 1.** Sociodemographic characteristics of the sample and correlates of being an active decliner.

	Characteristics of the total sample (n = 543)	Proportion of active decliners in each demographic subgroup (n = 117)	Unadjusted odds ratio for being an active decliner	Adjusted odds ratio for being an active decliner++
	n (column %+)	n (row %)	OR (95% CI)	OR (95% CI)
Age (years)				
25–34	188 (34.6)	21 (11.2)	1.00	1.00
35–44	159 (29.3)	25 (15.7)	1.48 (0.80–2.77)	1.15 (0.58–2.27)
45–54	115 (21.2)	22 (19.1)	1.88 (0.98–3.60)	1.44 (0.71–2.94)
55–64	81 (14.9)	49 (60.5)	12.18 (6.45–23.00)***	7.46 (3.58–15.53)***
Social grade				
AB: managerial/professional	84 (15.5)	17 (20.2)	1.00	
C1: supervisory	154 (28.4)	35 (22.7)	1.16 (0.60–2.23)	
C2 skilled manual	108 (19.9)	18 (16.7)	0.79 (0.38–1.64)	
D: semi-skilled/unskilled manual	106 (19.5)	23 (21.7)	1.09 (0.54–2.21)	
E: casual/lowest grade workers	91 (16.8)	24 (26.4)	1.41 (0.70–2.87)	
Ethnicity				
White British/Irish	390 (72.2)	87 (22.3)	1.00	
Any other White	52 (9.6)	12 (23.1)	1.05 (0.53–2.08)	
South Asian	52 (9.6)	8 (15.4)	0.63 (0.29–1.40)	
Black	29 (5.4)	6 (20.7)	0.91 (0.36–2.30)	
Mixed/other	17 (3.1)	2 (11.8)	0.46 (0.10–2.07)	
Working status				
Working full-time	179 (33)	40 (22.3)	1.00	
Working part-time	141 (26)	24 (17.0)	0.71 (0.41–1.25)	
Not working	223 (41.1)	53 (23.8)	1.08 (0.68–1.73)	
Marital status				
Married	329 (60.6)	50 (15.2)	1.00	1.00
Single	143 (26.3)	37 (25.9)	1.95 (1.21–3.15)**	2.44 (1.39–4.28)**
Previously married+++	71 (13.1)	30 (42.3)	4.08 (2.34–7.14)***	3.54 (1.79–7.03)***
Previous screening status				
Ever been screened	360 (66.3)	83 (23.1)	1.00	
Never been screened	181 (33.3)	34 (18.8)	0.77 (0.49–1.21)	

+ Due to rounding up or down, percentages may not add up to 100%.

++ Adjusted analyses includes all sociodemographic characteristics significant in unadjusted analyses; **p < 0.01, ***p < 0.001.

+++ Includes women who are separated, divorced, and widowed.

### Endorsement of barriers to screening

The proportion of women endorsing each of the barriers to screening is presented in Table 2. All women endorsed at least one of the 16 predefined barriers or an ‘other’ barrier (n = 543). Most women endorsed just one (n = 465), 51 women endorsed two, and 27 women endorsed three or more barriers. Active decliners were significantly more likely than intenders to report two (15% versus 8%, p = 0.01) or three or more barriers (9% versus 4%, p = 0.03). Just over 70% of women (n = 395) endorsed at least one of the 16 predefined barriers to screening. The most frequently endorsed barriers were being too busy to go for screening (16%), low relevance due to sexual behaviour (15%), embarrassment (12%), not having been invited to screening (12%), fear of what the test might find (10%), concerns about a man carrying out screening (8%), and having had a bad experience of screening in the past (8%). In addition to the 16 predefined barriers, 27% of women reported an ‘other’ barrier (n = 149). Most of these (70%) were unspecific, with no reason provided (e.g. ‘none apply’, ‘no reason’), 15% reported that they would or did go for screening, and 15% reported various miscellaneous barriers. Intenders were more likely to report an ‘other’ barrier than active decliners (31% versus 15%, p = 0.001).

**Table 2.** Odds of active decliners reporting each barrier to screening (compared with intenders).

	Proportion endorsing each barrier n (%)			Unadjusted odds ratio for being an active decliner	Adjusted odds ratio for being an active decliner+
	AllN = 543	IntendersN = 426	Active declinersN = 117		
I am too old to go for screening	22 (4.1)	12 (2.8)	10 (8.5)	3.22 (1.36–7.66)**	2.04 (0.73–5.71)
I have other more important things to worry about than screening	24 (4.4)	10 (2.3)	14 (11.9)	5.65 (2.44–13.09)***	5.96 (2.21–16.1)***
I am too busy to go for screening	89 (16.4)	74 (17.4)	15 (12.8)	0.70 (0.39–1.27)	
Low perceived relevance based on sexual behaviour	82 (15.1)	50 (11.7)	32 (27.4)	2.83 (1.71–4.68)***	3.07 (1.66–5.68)***
I am too embarrassed to go for screening	63 (11.6)	48 (11.3)	15 (12.7)	1.16 (0.62–2.15)	
I’m frightened of what the test might find	52 (9.6)	45 (10.6)	7 (5.9)	0.54 (0.24–1.23)	
I wouldn’t want a man to carry out the screening test	44 (8.1)	34 (8)	10 (8.5)	1.08 (0.52–2.25)	
I wouldn’t want anyone to know I had been for screening	12 (2.2)	9 (2.1)	3 (2.5)	1.22 (0.33–4.58)	
I’ve had a bad experience of screening in the past	44 (8.1)	28 (6.6)	16 (13.6)	2.25 (1.17–4.32)*	1.87 (0.87–4.02)
I have weighed up the risks and benefits and decided it’s not worth me going for screening	19 (3.5)	4 (0.9)	15 (12.7)	15.52 (5.04–47.74)***	11.53 (3.19–41.70)***
I haven’t been invited to cervical screening	66 (12.2)	61 (14.3)	5 (4.3)	0.27 (0.11–0.68)**	0.30 (0.10–0.91)*

+Adjusting for sociodemographic factors (age, marital status) and all significant barriers; *p < 0.05, **p < 0.01, ***p < 0.001.

Univariate logistic regression analyses were used to explore the association between endorsing each barrier and being an active decliner. Being an active decliner was associated with increased odds of endorsing the barriers ‘I am too old to go for screening’ (endorsed by 9% of active decliners versus 3% of intenders), ‘I have other more important things to worry about than screening’ (12% versus 2%), ‘I’ve had a bad experience of screening in the past’ (14% versus 7%), ‘I have weighed up the risks and benefits and decided it’s not worth me going for screening’ (13% versus 1%), and at least one barrier included in ‘low relevance due to sexual behaviour’ (27% versus 12%). Active decliners were at decreased odds of endorsing the barrier ‘I haven’t been invited to cervical screening’ (4% versus 14% of intenders).

Age, marital status, and the six barriers to screening that were significantly associated with being an active decliner were entered into a multivariate logistic regression model (see Table 2). After adjusting for age and marital status, endorsing the barriers ‘I am too old to go for screening’ and ‘I’ve had a bad experience of screening in the past’ were no longer significantly associated with being an active decliner; however, endorsing ‘I have other more important things to worry about than screening’ remained significant (p < 0.001), as did ‘I have weighed up the risks and benefits and decided it’s not worth me going for screening’ (p < 0.001), ‘I haven’t been invited to cervical screening’ (p = 0.033), and endorsing at least one barrier related to ‘low relevance due to sexual behaviour’ (p < 0.001).

### Interest in using self-sampling

**Figure 1.**
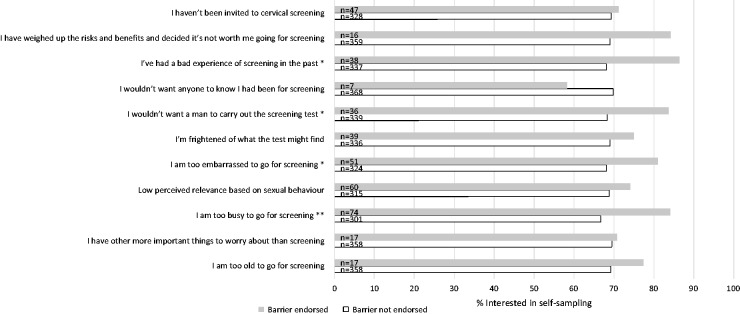
Interest in self-sampling by endorsement of each barrier to screening.*p < 0.05, **p < 0.01.

Of the intenders, 70% reported that they would prefer to do the test themselves at home (47% ‘definitely’ and 23% ‘probably’). Of the active decliners, 66% reported that they would prefer to do the test themselves at home (43% ‘definitely’ and 23% ‘probably’). There was no difference in overall preference for self-sampling between intenders and active decliners (χ²(1, N = 539)=0.281, p = 0.60). Univariate logistic regression analyses were used to explore whether a preference for self-sampling was associated with endorsement of specific barriers to screening (see Figure 1). Endorsement of four barriers was significantly associated with greater preference for self-sampling: ‘I am too busy to go for screening’ (OR = 2.63, 95% CI: 1.44–4.82), ‘I wouldn’t want a man to carry out the test’ (OR = 2.38, 95% CI: 1.04–5.47), ‘I’ve had a bad experience of screening in the past’ (OR = 2.97, 95% CI: 1.23–7.17), and ‘I am too embarrassed to go for screening’ (OR = 1.99, 95% CI: 1.03–3.85).

## Discussion

This study explored differences in self-reported barriers to cervical screening among women who had formed intentions about attending screening in the future, comparing women who intended to attend for screening with those who had decided not to. Women in both groups endorsed a wide range of barriers, but we identified some barriers that were more commonly reported by active decliners.

In line with previous research,^13,14^ women who had decided not to be screened were twice as likely to endorse barriers relating to ‘low perceived relevance based on sexual behaviour’. While women may feel they are less at risk of developing cervical cancer because of their current sexual behaviour, it has been suggested that it can take between 10 and 20 years for cervical cancer to develop from an HPV infection,^27^ so a woman’s current sexual behaviour does not necessarily reflect her current risk. Efforts should be made to ensure all women have access to relevant risk information to enable a fully informed choice. This might include clarifying common misconceptions about the link between sexual behaviour and HPV, and emphasizing that any sexual contact could expose a woman to HPV, a woman could have HPV for many years without knowing it, and it could take many years for cervical cancer to develop. This information could be added to the information leaflet that is sent to all women who are invited for NHS cervical screening.

Women who had decided not to be screened were also more likely to endorse the barrier ‘I have other more important things to worry about’ than women who were intending to be screened. Previous findings have shown that having other things to worry about is a key reason why women choose not to attend cervical screening, as long as they feel healthy.^14^ While women who had decided not to be screened were more likely to say that they had weighed up the risks and benefits of screening, this statement was only endorsed by 13% of active decliners. Deciding not to attend screening is a legitimate choice, but it is important that women consider the risks and benefits of screening first, and base their decision on accurate information. The use of decision aids to improve informed decision making has been found to be promising in breast and colorectal cancer screening, but more research is needed to explore their use in the cervical screening context.^28^

Previous studies have suggested some commonly described attitudes to screening, including being embarrassed, concerned about the test, and concerned about the gender of the smear taker, do not directly influence attendance.^4,29^ We also found that this was the case when comparing those who have made different decisions about screening, with the barriers ‘I am too embarrassed to go for screening’, ‘I wouldn’t want a man to carry out the screening test’, ‘I wouldn’t want anyone to know I had been for screening’, and ‘I’m frightened of what the test might find’ endorsed by a similar proportion of intenders and active decliners.

Most participants reported that they would ‘definitely’ or ‘probably’ prefer home-based self-sampling, and interest in self-sampling was particularly high among those who were too busy or too embarrassed to go for screening, wouldn’t want a man to carry out the test, or had had a bad experience of screening in the past. Offering HPV self-sampling to screening non-attenders, either by post, or opportunistically in primary care, could be an effective strategy for increasing participation among women who have decided not to attend. A study conducted in the United Kingdom with persistent non-responders (women who had not responded to at least two invitations to attend for cervical screening), randomized women to be posted either an HPV self-sampling kit or a third invitation.^30^ In the self-sampling group 10% returned their kit, and in the group sent an additional invitation, 5% attended screening.^30^ Another study in the United Kingdom offered self-sampling to cervical screening non-attenders opportunistically when they attended a primary care appointment, and found that 9% returned a self-sample.^31^

Our study benefits from using population-based data to identify women who have made a decision about whether to attend for cervical screening in the future. The data were based on self-reported screening history, and while this method is commonly used, participants may have overestimated screening utilization.^32^ In addition, some of the barriers to screening were endorsed by small numbers of participants, and therefore the study may not have been adequately powered to detect differences between groups. The barrier ‘I haven’t been invited to cervical screening’ was one of the most frequently endorsed across all age groups, with most participants (61/66) who endorsed this barrier reporting that they were intending to attend screening in the future. It is not clear why over 10% of participants in our sample reported that they had not received an invitation to be screened, as eligible women should automatically be invited if they are registered with a GP. Very few women reported that they were not registered with a GP (n = 10). Written invitations sent by post are becoming a less common method of communication,^33^ and some women may have received the invitation but not read it. It is important that all women are made aware that only those registered with a GP will be invited to take part in screening. Another limitation is that we did not have comparative data from women who were up to date with cervical screening.

## Conclusions

To our knowledge, this is the first study to compare barriers to participation in cervical screening between women who have made an active decision not to be screened in the future and women who are intending to take part. Women who actively decline screening tend to be older, unmarried, and perceive screening as being of low personal relevance. HPV self-sampling could address specific emotional and practical barriers and is likely to be an acceptable alternative for screening non-attenders who have not been screened in the conventional way.
